# Assessment of Dietary Intake Using Food Photography and Video Recording in Free-Living Young Adults: A Comparative Study

**DOI:** 10.1016/j.jand.2020.09.040

**Published:** 2021-04

**Authors:** Rouba Naaman, Alison Parrett, Daliah Bashawri, Inès Campo, Katie Fleming, Ben Nichols, Elizabeth Burleigh, Janice Murtagh, James Reid, Konstantinos Gerasimidis

**Keywords:** Dietary assessment, Food photography, Video recording, Weighed food records, Image-based dietary assessment

## Abstract

**Background:**

Conventional methods of dietary assessment are prone to recall bias and place burden on participants.

**Objective:**

Our aim was to compare the performance of image-based dietary assessment (IBDA), including food photography (FP) and video recording (VR), with the criterion of weighed food records (WFR).

**Design:**

In this comparative study, participants captured meals using FP and VR before and after consumption, over 2 days. Food type and portion size were assessed using the images and videos. Energy and nutrient intakes (mean of 2 days) were compared against WFR.

**Participants/settings:**

Eighty-four healthy adults (mean [standard deviation] age = 29 [8] years), recruited through advertisement in Glasgow, UK, between January and August 2016 were enrolled in the study. Eighty participants (95%) (mean [standard deviation] age = 28 [7] years) completed the study and were included in the analysis.

**Main outcome measures:**

Agreement in estimated energy and nutrient intake between WFR and IBDA. The IBDA method feasibility was evaluated using a questionnaire. Inter-rater and intra-rater reliability were assessed.

**Statistical analysis performed:**

The performance of the IBDA methods against WFR and their inter and intra-rater reliability were tested with Bland-Altman plots and Spearman correlations. Intra-class agreement between methods was assessed using κ statistics.

**Results:**

Inter-rater reliability was strong for both IBDA methods in estimating energy intake (ρ-coefficients: FP = 0.80; VR = 0.81). There was no difference in the agreement between the 2 assessors. Intra-rater reliability was high. FP and VR underestimated energy intake by a mean (95% agreement limits) of –13.3% (–56.4% and 29.7%) and –4.5% (–45.5% and 36.4%), respectively. IBDA demonstrated moderate-to-strong correlations in nutrient intake ranking, median ρ-coefficients for all nutrients: FP = 0.73 (interquartile range, 0.09) and VR = 0.82 (interquartile range, 0.02). Inter-class agreement of IBDA methods was moderate compared with the WFR in energy intake estimation. IBDA was more practical and enjoyable than WFR.

**Conclusions:**

IBDA and VR in particular demonstrated a moderate-to-strong ability to rank participants’ dietary intake, and considerable group and inter-class agreement compared with the WFR. However, IBDA was found to be unsuitable for assessment in individuals.

**Research Snapshot**

**Research Question:** How do image-based dietary assessment methods compare with weighed food records in estimating the intake of free-living young adults?

**Key Findings:** Food image photography and, in particular, video recording, presented considerable group and inter-class agreement compared with the criterion of weighed food records, but they were found to be unsuitable for assessment in individuals.

Several methods have been developed to assess the food and nutrient intake of individuals and populations, including weighed food records (WFR), food frequency questionnaires, and 24-hour recalls.^1^^,^^2^ The method of choice depends on the accuracy, precision, type of food or nutrient analysis required, population characteristics, available resources, and burden imposed on participants and assessors.^3^ Some of these methods, like 24-hour dietary recalls, are memory dependent and prone to recall bias, whereas others require meticulous recording and weighing or estimation of food portion size; which places a considerable burden on participants.^4^^,^^5^ As a result, over- or underestimation of food portion sizes, misreporting of intake, and distortion of regular eating habits often invalidate the outcomes of conventional dietary assessment.^6^^,^^7^

The use of digital technology in the process of dietary intake assessment has been suggested as a way to overcome inherent limitations of conventional approaches.^8^ Previous studies tested the performance of web-based, self-administered 24-hour recalls and food frequency questionnaires.^9^, ^10^, ^11^ Although a minimum bias was reported by using the electronic versions of these questionnaires, it was shown that electronic questionnaires were time-consuming and placed a burden on the respondents by asking them to navigate large databases of food items and to estimate their portion size.^12^ The use of personal digital assistants early in the millennium simplified the process of dietary assessment, however, limitations were reported^13^^,^^14^ with regard to the complexity of food intake entry and difficulty writing and navigating on small screens, which increased burden on less educated and older individuals.^15^^,^^16^ Use of smartphone applications in assessing dietary intake has gained popularity.^17^ It has been reported that use of smartphone applications might be a useful, practical, and more enjoyable alternative to completing 24-hour recalls in epidemiologic studies.^18^

As a result of high accessibility, reduced cost, and convenience of cameras and smartphones, growing interest has been expressed among researchers in using image-based dietary assessment (IBDA) in free-living individuals^19^^,^^20^ and controlled environments, such as hospitals,^21^^,^^22^ laboratories,^19^^,^^23^ and cafeterias.^8^ IBDA refers to methods that use photos or videos of foods and drinks to describe dietary intake.^24^ IBDA can be active, by asking individuals to capture the pictures,^13^^,^^20^^,^^25^ or passive, by using wearable cameras that capture pictures of the consumed foods in real time.^23^ IBDA methods are often combined with food records or voice recording describing served and consumed meals.^20^^,^^26^ Previous research showed that IBDA made dietary intake assessment much easier than using traditional methods.^24^ It minimized recall bias and reduced the respondents burden during the recording period, allowing rapid and easy collection of food intake data.^19^

The use of video recording (VR) may provide additional benefits to other IBDA methods. Recording food video footage allows the individuals to describe foods and meals in a multidimensional plane, as well as the time and place the meal or food was consumed. A recent study compared the use of the VR method with WFR for assessing the dietary intake of 30 adults in a cafeteria setting.^27^ The study reported that VR was a valid method for estimating dietary intake in this population and setting. The performance of VR has not yet been tested in free-living conditions, such as at home, at work, or when dining out.

The aim of this study was to compare the performance of IBDA, including the use of digital food photography (FP) and VR with the criterion method of WFR in free-living young adults.

## Materials and Methods

### Participants Recruitment

A convenience sample of adults was recruited from Glasgow, UK, between January and August 2016. Participants were recruited through social media pages (Facebook, University of Glasgow internal social network, and Gumtree), and via printed advertisements on notice boards located at the University of Glasgow campus and in public areas around Glasgow city (eg, train stations, supermarkets, and coffee shops). Participants 18 years or older were eligible to participate, regardless of social and occupational background and ethnicity. Participants were excluded if they were following a restricted diet due to any medical reason during the study period. Pregnant women were not included. Participants' age, sex, ethnicity, and profession were self-reported. Participants were weighed once wearing light clothing without their shoes using a digital scale (EKS) to the nearest 0.1 g. Height was measured to the nearest 0.1 cm using a portable stadiometer (Leicester Height Measure) and these measurements were used to calculate body mass index (BMI). BMI was classified according to the Centers for Disease Control and Prevention^28^ cutoffs. The study protocol was approved by the College of Medical, Veterinary and Life Sciences Ethics Committee at the University of Glasgow (project 200150034). All participants signed informed consent. Each participant received a £20 ($25) voucher incentive.

### Dietary Intake Assessment Using WFR

Participants were provided with paper-based food recording diaries and kitchen scales (Salter) and were instructed to record the type and to weigh (in grams) all food and drink items consumed during the recording period. Food and drink items were weighed before consumption and after each meal if any was leftover. As the primary aim of the study was to evaluate the validity of IBDA and not to assess participants’ habitual dietary intake or inter-daily variation, study participants were asked to record their intake over 2 days that were not necessarily consecutive; 1 of which was a weekend day to compare the performance of the IBDA methods between week and weekend days.^29^ Participants were asked to weigh all food and drinks consumed, including main meals and snacks and those consumed at home and outside the home, such as at work, college, or restaurants. For homemade dishes, recipe ingredients, method of cooking, and the number of people the recipe served were recorded. For takeout meals or dishes not prepared at home, such as those consumed at a restaurant or a friend’s house, participants were instructed to weigh their meals with the portable scales provided and to describe their meals in the diary in as much detail as possible. Participants were asked to record product brand names in the diary, which was used to elicit more information about portion size from manufacturer’s websites. When food weighing was not possible, participants were instructed to use household measures, such as teaspoons, tablespoons, or cups, and food label information. Participants were asked to keep to their usual eating habits during the recording period. A WFR instruction leaflet was provided to the participants with an example of a 1-day dietary record to demonstrate to them how to complete their diaries. Participants were asked to record meals with the IBDA methods before completing the WFR.

### Dietary Intake Assessment Using IBDA

Over the same 2 days, participants were asked to take digital pictures and record video footage of all food and drinks consumed, before and after each meal, using a digital camera provided. There was no specified order of which IBDA method to use first when dietary intake was recorded, as this would not affect the downstream dietary analysis performed by the independent assessor. However, both IBDA methods were completed before the WFR.

**Figure 2:**
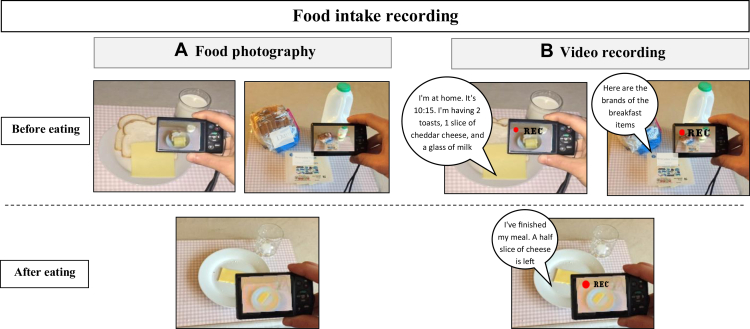
Process of food intake assessement using food photography and video recording in free-living adults over 2 days in Glasgow, UK, between January and August 2016. Participants recorded their food intake using a provided digital camera held at a camera angle of 45 degrees and at a 1-arm distance from the meal. They captured their intake before and after eating and in addition to recording the brand and the labels of their food. (A) In food photography method, participants captured pictures for their meals. (B) In video recording, participants recorded videos of their meals and described their meals verbally.

Participants were instructed how to use the camera and to record videos. Training examples were provided with written instructions (Figure 1; available at www.jandonline.org). They were asked not to include themselves in the recorded pictures and videos and not to use a diary or notes to record any additional information. All participants were trained by the study researchers to use the camera before the days of dietary recording. Participants captured pictures and recorded videos of their meals with a digital camera (Canon, IXUS 160). Fiduciary markers were not used in the images, as these may have increased inconvenience and burden to participants. Instead, for the IBDA method, participants were instructed to hold the camera at an angle of 45 degrees and at a 1-arm distance from the object (Figure 2). Participants were asked to capture pictures of their meals before and after eating. They were told to take as many pictures and videos of each meal as possible and wished. If they were eating a prepackaged meal, they were also asked to take pictures of the brand, portion size, and the whole food label, including the ingredient list and the back-of-package Nutrition Facts label. If participants were having homemade dishes, they were asked to photograph the recipe ingredients used, ingredients food labels, the final meal produced, and the portion size served.

Likewise, for the VR method, participants were instructed to record videos of their meals before eating and to record another video after eating to show whether there were any leftovers. While recording video, they were asked to describe verbally the food they were having in as much detail as possible. The place and time of the meals were reported, then the type, brand name, and size of the plate or size of serving were described (eg, a medium serving of pasta with 3 tablespoons of Bolognese sauce on an average size shallow plate). Lastly, the cooking method (eg, fried, grilled, or baked), and any condiments or flavorings (eg, spreads, sugar, sauces, pepper, and salt) were recorded. If they had homemade dishes, the name of the recipe, ingredients, number of people the recipe served, cooking method, and how much of the whole recipe they were served and eaten were recorded. When eating outside their home, they were asked to record as much detail as possible, including the place and type of food and provide a verbal description of food portion sizes using household measures, qualitative estimates of meal size (eg, small or medium), and food label information.

### Methods Feasibility and Practicality Evaluation

At study completion, participants evaluated the feasibility and practicality of IBDA methods. A validated questionnaire was not used per se, as such a questionnaire was not available. Instead, this study surveyed the participants’ opinions and asked for feedback about the practicality of the study procedures and the dietary assessment methods using a paper-based survey. This structured survey was based on questions agreed on by the senior members of the research team and relevant to the primary objectives of the study. IBDA and WFR were assessed in 6 domains, including the impact on social interactions, level of difficulty, time needed to complete food intake recording, whether the method was monotonous, and the level of enjoyment and practicality. Assessment was done by visual analogue scale for each domain, which consisted of a 10-cm long blank line, labeled with the extreme answers “not at all” at the left end and “very much” at the right end, and a mark at the middle of line. Participants were requested to place a vertical mark on the lines to indicate their opinion about the study methods. A score was recorded based on the distance of their mark from the left-hand side (0) where “not at all = 0” and “very much = 10.” In addition, participants were asked which IBDA method they found more difficult to apply. They were also asked whether they changed the quantity of food they consumed during the recording period from their normal consumption, whether they refrained from recording all food items, or whether they changed their usual eating pattern during the recording period and the reason behind this. The participants had the opportunity to provide additional comments.

### Energy and Nutrient Intake Analysis

A research dietitian (R.N.) received training in coding by senior members of the nutrition academic team, including 1 senior academic dietitian (K.G.). The research dietitian associated with the study viewed the collected meal pictures and recorded videos on a computer screen for analysis. The analysis was done based on estimation of the type and portion size of the meals using the collected food pictures or video footage only. For each IBDA, analysis of the collected pictures and videos was done separately by performing the following 2 main steps: identification of the type of food items recorded in both pictures and videos and estimation of food items portion size in grams. This was done using a stepwise methodology. Firstly, using information from food packaging and food labels recorded in participants' pictures for the FP analysis or video footages for VR analysis. If no food packaging or labels were recorded, the portion size was estimated using the *Food Photographic Atlas of Food Portion Sizes*.^30^^,^^31^ This atlas consists of 78 food items commonly consumed in the United Kingdom. Each food item is shown in a series of 8 photographs that reflect different portion sizes. Using the food atlas, 1 of the 8 photographs of the appropriate food item was chosen as representing the closest match in portion size to each component of patients' meal pictures. The amount of liquid consumed, such as milk, tea, coffee, and juices, were estimated using the additional guides of household measures in the food atlas. If the food item was not available in the food atlas, the food portion size guide of *WinDiet*s software, version 2010,^32^ or the *Food Portion Sizes* booklet from the UK Food Standards Agency^33^ were used. For food items for which the researchers were unable to estimate portion size using the aforementioned approach, information was collected from the product manufacturer or restaurant website. When this information was not available, the researchers visited 2 mainstream UK supermarkets (Tesco and Sainsbury's), which provide online Nutrition Facts labels for the most common grocery items sold in UK supermarkets.

To minimize portion-size estimation bias and to reduce the bias of information transmission from one method to another, data collected by each IBDA method were analyzed separately and not at the same time as the analysis of the other methods for the same participants. The researcher started with analysis of all collected food pictures for all participants, followed by analyzing all collected videos for all participants and then all data collected by the WFR for all participants in a random order. For each assessment day, pictures that were recorded before consumption were analyzed first in terms of type of food items, brand names, and portion sizes, and then those recorded after intake (ie, leftovers). Similarly, when videos and WFR were analyzed, the same order was applied. If no leftovers pictures or videos were recorded, it was assumed that the participant consumed the entire food portion and, in that case, the leftover portion size was estimated as zero. Participants were asked to capture several pictures and videos of the same meal or food item. Pictures and videos with the best quality and clarity for the same meal or food item were chosen for analysis and, when needed, more than 1 photo or video was used to elicit additional information. Unusable photos or videos were discarded.

The amount of consumed food items and drinks was calculated as the difference between the estimated portion served and estimated amount of leftovers. Dietary analysis was performed with *WinDiets* software, version 2010,^32^ for energy, macronutrients, and selected micronutrients.

Energy intake underreporting was assessed by calculating the 2-day average ratio of energy intake measured by the WFR over the basal metabolic rate of the participant. The basal metabolic rate was calculated using the Schofield equation^34^ based on sex, age, and weight. Underreporting cutoff value was calculated using the following Goldberg equation^35^:

$$EI\;:\;BMR>PAL\;\times \exp\;\left[SD_{min}\times \;\frac{S/100\;}{\surd n}\right],$$

where PAL of 1.4 represents the physical activity level of participants with light activity levels. SD_min_ is equal to –2 (95% CI), n is 1 for individual evaluation.

$$S=\sqrt{\frac{CV_{wEI}^{2}}{d}+CV_{wB}^{2}+CV_{tP}^{2}}$$

Based on Black standard coefficients,^36^ where *CV*_*wEI*_ is equal to 23, which represents the within-subject coefficient of variation in energy intake, *d* is equal to 2, which represents the number of dietary intake recording days, *CV*_*wB*_ is equal to 8.5, which represents the within-subject coefficient of variation in repeated basal metabolic rate measurements and *CV*_*tP*_ is equal to 15 the between-subject coefficient of variation in PAL. From this equation *S* is equal to 23.7 and participants with a value of <0.872 were considered as underreporters of energy intake.

### Inter-Rater and Intra-Rater Reliability

Using the RAND() function in Microsoft Excel,^37^ IBDA food pictures and video footage from a random sample of 30 single days from 30 participants participants, initially analyzed by R.N., were reanalyzed by another trained research dietitian (D.B.) to assess inter-rater reliability. Likewise, IBDA-captured food pictures and video footage for 20 randomly selected days from 20 randomly selected participants were reanalyzed within 3 years from the initial analysis to evaluate intra-rater reliability.

### Statistical Analysis

Statistical analysis was undertaken using *Minitab* statistical software, version 16,^38^ *MedCalc* software, version 15.8^39^ and in *R*, version 3.4.0.^40^ Inter-rater and intra-rater reliability were assessed using Spearman rank correlation. Spearman rank correlations were used to test the ability of IBDA to rank participants’ dietary intake like with the WFR. Paired *t* test was applied to test the mean estimation error (ie, accuracy) between the methods. Inter-individual agreement (ie, precision) between the IBDA methods and WFR, as well as the IBDA methods’ inter-rater and intra-rater reliability were assessed with Bland-Altman plots.^41^ The difference in estimation error between weekdays and weekend days was assessed with paired *t* test. Weighted κ analysis was used to assess inter-class agreement in estimating energy and macronutrients intake in tertiles between the IBDA and WFR. For each participant the average dietary intake of the 2 days was calculated except for the subanalysis for difference in estimation error between week and weekend days. *P* < 0.05 was statistically significant. For interpretation of κ values, the classification by Landis and Koch^42^ was used. Likewise, for correlation analysis the classification by Schober and colleagues was applied.^43^

### Power Calculation

According to Cade and colleagues,^44^ a sample size larger than 50 is required when Bland-Altman plots are used to assess the agreement between 2 methods and estimation of 95% limits of agreement is required.

## Results

### Participants Characteristics

From 89 participants who expressed an interest in participating in the study, 1 did not meet the study inclusion criteria and 4 others did not agree to participate. From the 84 participants (mean [standard deviation] age = 29 [8] years) who provided informed consent, 80 participants (male, n = 25 [31%]; mean [standard deviation] age = 28 [7] years) completed the study with a total of 160 dietary assessment days. More than one-half of participants (55%) were White, students (81%), and health professionals (46%). Fifty-five (69%) had a normal BMI, 16 (20%) were overweight, and 7 (9%) were obese (Table 1). From the total number of pictures or videos captured, 2% of videos and 4% of images were not useable due to poor resolution and were discarded. Mean estimated energy intake of the study participants was 1,600 kcal/day and 31 participants (39%) underreported their energy intake.

**Table 1:** Characteristics of study participants in a study comparing the use of image-based dietary assessment against weighed food records for assessing dietary intake in 80 free-living adults (n = 80) in Glasgow, UK, between January and August 2016

Characteristics	Data
**Age, y, mean (SD**^a^**)**	28 (7)
**Weight, kg, mean (SD)**	67 (14)
**Height, cm, mean (SD)**	167 (87)
**BMI,**^b^**mean (SD)**	23.7 (3.6)
**Sex, n (%)**	
Male	25 (31)
Female	55 (69)
**BMI classification,**^c^**n (%)**	
Underweight (<18.5)	2 (2)
Normal (18.5 to <25)	55 (69)
Overweight (25 to <30)	16 (20)
Obese (≥30)	7 (9)
**Ethnicity, n (%)**	
White	44 (55)
Asian	27 (34)
Other^d^	9 (11)
**Student status, n (%)**	
Student	65 (81)
Nonstudent	15 (19)
**Occupation, n (%)**	
Health professionals	37 (46)
Science and engineering	30 (38)
Business and social science	13 (16)

a SD = standard deviation.

b BMI = body mass index.

c Body was classified is based on the Centers for Disease Control and Prevention BMI categories.^28^

d Other ethnicities include Arab, Hispanic, and Mexican.

### Inter-Rater Reliability

Estimates of energy (FP: ρ = 0.80 and VR: ρ = 0.81) and carbohydrate intake (FP: ρ = 0.84 and VR, ρ = 0.83) were strongly correlated between the 2 assessors. Moderate-to-strong correlations were observed for the intakes of protein (FP: ρ = 0.69 and VR: ρ = 0.63) and fat (FP: ρ = 0.65 and VR: ρ = 0.78). Using Bland-Altman analysis, the mean difference in estimation of energy intake between the 2 assessors was –35 kcal (mean difference: *P* = 0.525; 95% limits of agreement –1,060 and 989 kcal) for WFR, 33 kcal (mean difference: *P* = 0.517; 95% limits of agreement –935 and 1,000 kcal) for FP and –77 kcal (mean difference: *P* = 0.117; 95% limits of agreement –1,061 and 908 kcal) for VR. None of these differences achieved statistical significance.

### Intra-Rater Reliability

Intra-rater reliability in estimating the intake of energy using the VR and the FP was very strong (FP: ρ = 0.83 and VR: ρ = 0.87). Both methods showed strong intra-rater reliability in estimating the intake of carbohydrates (FP: ρ = 0.77 and VR: ρ = 0.79), protein (FP: ρ = 0.72 and VR: ρ = 0.74), and fat (FP: ρ = 0.73 and VR: ρ = 0.75). Based on the Bland-Altman plots, the mean difference in estimation error of energy intake was 46 kcal (mean difference: *P* = 0.415; 95% limits of agreement –438 and 530 kcal) for FP and 23 kcal (mean difference: *P* = 0.570; 95% limits of agreement –326 and 372 kcal) for VR.

### Rank Correlation Analysis of IBDA with WFR

Spearman rank correlation coefficients between the IBDA methods and the WFR are shown in Table 2. For both IBDA methods, estimated nutrient intakes significantly correlated with those measured by WFR (*P* < 0.001). Correlation coefficients ranged from ρ = 0.60 to 0.88 and were generally >0.7 (median = 0.73; interquartile range, 0.09) and ranged from ρ = 0.74 to 0.90 and were generally >0.8 (median = 0.82; interquartile range, 0.02) with the use of FP and VR, respectively (Table 2).

**Table 2:** Spearman rank correlation coefficients between weighed food records and the image-based dietary assessment methods in assessing daily energy and nutrients intake in free-living adults in Glasgow, UK, between January and August 2016

Nutrients	FP^a^ (n = 80)	VR^b^ (n = 80)	VR^c^ (n = 75)
	←*ρ-coefficient*∗∗∗→		
Energy, kcal	0.78	0.81	0.84
Carbohydrates, g	0.74	0.80	0.86
Protein, g	0.69	0.81	0.79
Fat, g	0.70	0.82	0.84
Saturated fat, g	0.71	0.81	0.85
Polyunsaturated fat, g	0.64	0.86	0.84
Monounsaturated fat, g	0.60	0.76	0.79
Sugars, g	0.82	0.82	0.90
Starch, g	0.65	0.82	0.85
Nonmilk extrinsic sugar, g	0.62	0.81	0.85
Nonstarch polysaccharide, g	0.78	0.85	0.88
Vitamin A, μg	0.81	0.82	0.82
Thiamine, mg	0.81	0.81	0.80
Vitamin D, μg	0.68	0.82	0.84
Vitamin E, mg	0.73	0.82	0.77
Vitamin C, mg	0.75	0.77	0.84
Folate, μg	0.88	0.81	0.82
Vitamin B-12, μg	0.63	0.76	0.84
Dietary fiber, g	0.84	0.90	0.89
Calcium, mg	0.73	0.85	0.88
Potassium, mg	0.73	0.74	0.80
Iron, mg	0.73	0.82	0.82
Magnesium, mg	0.74	0.75	0.79
Zinc, mg	0.78	0.83	0.82
Selenium, μg	0.74	0.84	0.83

a FP = food photography.

b VR = video recording.

c VR = video recording for participants without missing food items.

∗∗∗ *P* < 0.001.

### Mean and Inter-Individual Agreement Between IBDA with WFR

**Figure 3:**
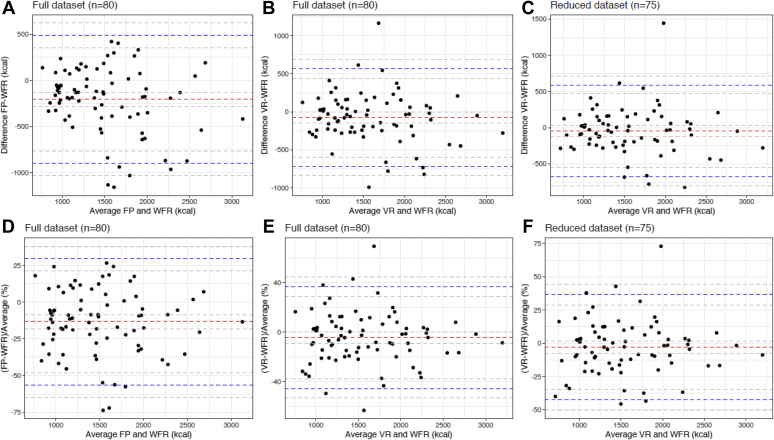
Bland-Altman plots with 95% limits of agreement between weighed food records (WFR) and (A) food photography (FP), (B) video recording (VR), and (C) video recording excluding days with missing food items for estimating actual energy intake, and between WFR and (D) FP, (E) VR, and (F) video recording excluding days with missing food items for estimating percentage of energy intake in 80 free-living adults over 2 days in Glasgow, UK, between January and August 2016. Red dashed lines are the mean estimation differences between the 2 methods, blue dashed lines represent the upper and lower 95% limits of agreement and gray dashed lines represent their associated 95% CIs.

The agreement in energy intake between the IBDA and the WFR is displayed in the Bland-Altman plot (Figure 3). Mean energy intake estimated by FP and VR was significantly lower by a mean of –207 kcal (mean difference: *P* < 0.001; 95% limits of agreement –902.1 and 487.9 kcal) and –76 kcal (mean difference: *P* = 0.04; 95% limits of agreement –716.2 and 564.5 kcal) respectively, compared with WFR. Hence, energy intake was underestimated by a mean of –13.3% (95% limits of agreement –56.4% and 29.7%) and –4.5% (95% limits of agreement –45.5% and 36.4%) for FP and VR, respectively.

The mean intakes of almost all nutrients estimated by the use of VR were significantly underestimated from the intakes measured by the WFR, except for the intake of protein, fat, saturated fat, monounsaturated fat, starch, nonstarch polysaccharide, vitamin D, vitamin B-12, dietary fiber, calcium, and selenium (Table 3). Using the FP method, the mean intake of almost all nutrients estimated by FP were significantly underestimated from that estimated by WFR, except for carbohydrates, nonmilk extrinsic sugar, vitamin D, vitamin C, and vitamin B-12 (Table 3).

**Table 3:** Mean differences with 95% limits of agreement between the weighed food records and the image-based dietary assessment methods for daily actual nutrients intake, percentage of nutrients intake, and percentage of total energy intake of macronutrients in free-living adults in Glasgow, UK, between January and August 2016

Nutrients	FP^a^ (n = 80)			VR^b^ (n = 80)			VR^c^ (n = 75)		
	Mean difference	Limits of agreement	*P* value^d^	Mean difference	Limits of agreement	*P* value^d^	Mean difference	Limits of agreement	*P* value^d^
Energy, kcal	–207	–902, 488	<0.001	–75.8	–716, 564	0.041	–44.6	–681.5, 592.2	0.238
Energy %	–13.3	–56.4, 29.7		–4.5	–45.5, 36.4		–2.9	–42.5, 36.6	
Carbohydrates, g	–12.0	–173.9, 149.7	0.194	–9.5	–92.2, 73.2	0.047	–5.9	–78.0, 66.0	0.163
Carbohydrates %	–9.1	–69.3, 50.9		–4.3	–47.9, 39.3		–2.2	–41.1, 36.5	
Protein, g	–6.9	–47.1, 33.2	0.003	–2.3	–36.7, 31.9	0.23	–1.2	–40.1, 37.7	0.599
Protein %	–9.2	–66.2, 47.6		–2.9	–48.9, 43.0		–1.7	–51.1, 47.6	
Fat, g	–10.9	–55.9, 33.9	<0.001	–3.1	–38.5, 32.3	0.129	–1.4	–34.9, 31.9	0.454
Fat %	–16.4	–80.5, 47.6		–4.7	–55.6, 64.1		–3.2	–52.5, 66.1	
Saturated fat, g	–2.6	–19.6, 14.3	0.007	–0.5	–13.7, 12.5	0.458	0.01	–15.3, 15.4	0.991
Saturated fat %	–12.7	–85.4, 59.9		–5.0	–63.8, 53.6		–3.6	–63.9, 56.5	
Polyunsaturated fat, g	–3.1	–17.0, 10.7	<0.001	–1.2	–11.5, 8.9	0.029	–1.1	–9.6, 7.3	0.028
Monounsaturated fat, g	–3.1	–18.7, 12.4	<0.001	–0.8	–12.8, 11.1	0.23	–0.7	–10.8, 9.3	0.232
Sugars, g	–7.9	–59.8, 44.0	0.009	–6.0	–56.0, 44.0	0.038	–2.5	–40.0, 34.9	0.248
Sugars %	–15.8	–80.2, 48.6		–12.2	–79.4, 54.9		–7.6	–64.1, 48.9	
Starch, g	–8.9	–61.7, 43.8	0.004	–1.7	–39.0, 35.4	0.406	–1.9	–38.6, 34.7	0.371
Nonmilk extrinsic sugar, g	–1.5	–41.1, 38.1	0.502	–3.0	–23.2, 17.0	0.009	–1.7	–17.8, 14.3	0.066
Nonstarch polysaccharide, g	–1.3	–7.0, 4.4	<0.001	–0.4	–5.5, 4.6	0.119	–0.2	–5.1, 4.6	0.415
Vitamin A, μg	–97.2	–845.5, 651.0	0.025	–87.0	–853.4, 679.3	0.05	–88.0	–902.7, 726.6	0.07
Thiamine, mg	–0.1	–0.7, 0.4	<0.001	–0.1	–0.8, 0.6	<0.001	–0.1	–0.9, 0.6	0.006
Vitamin D, μg	0.1	–3.8, 4.1	0.475	0.1	–3.8, 4.0	0.724	–0.02	–3.7, 3.6	0.914
Vitamin E, mg	–1.3	–8.1, 5.5	<0.001	–1.2	–9.7, 7.3	0.015	–1.2	–10.4, 7.9	0.025
Vitamin C, mg	–3.2	–86.2, 79.6	0.494	–9.7	–83.1, 63.5	0.022	–4.5	–52.0, 42.9	0.11
Folate, μg	–34.4	–172.5, 103.7	<0.001	–22.7	–163.7, 118.1	0.006	–18.2	–155.4, 118.9	0.027
Vitamin B-12, μg	0.2	–10.2, 10.8	0.656	–0.2	–2.8, 2.2	0.054	–0.2	–2.5, 2.0	0.041
Dietary fiber, g	–1.0	–8.4, 6.3	0.016	–0.3	–7.0, 6.3	0.383	–0.2	–7.2, 6.6	0.497
Calcium, mg	–92.7	–647.8, 462.3	0.004	–33.3	–480.8, 414.2	0.195	–24.3	–464.6, 415.9	0.351
Potassium, mg	–220.1	–1,308.1, 867.8	<0.001	–132.1	–1,057.6, 793.2	0.014	–110.0	–939.1, 719.1	0.027
Iron, mg	–1.9	–8.5, 4.6	<0.001	–1.0	–6.5, 4.4	<0.001	–0.8	–6.3, 4.6	0.01
Magnesium, mg	–25.0	–116.0, 65.9	<0.001	–12.8	–107.7, 82.0	0.02	–9.2	–96.0, 77.4	0.074
Zinc, mg	–0.6	–5.0, 3.7	0.015	–0.5	–3.9, 2.9	0.012	–0.4	–3.7, 2.9	0.038
Selenium, μg	–4.7	–33.8, 24.2	0.005	–1.5	–23.6, 20.6	0.233	–1.6	–24.2, 20.9	0.221

a FP = food photography.

b VR = video recording.

c VR = video recording for participants without missing food.

d *P* values were calculated by paired *t* test.

Intake of all nutrients estimated by the IBDA methods presented wide limits of agreement, suggesting high estimation error at assessments per participant. The narrowest of all, hence, the best precision at assessment per participant, was observed for the VR method (Table 3). There was no difference in the performance of the FP and the VR method on weekdays compared with weekend days (*P* > 0.05) (Table 4).

**Table 4:** Mean difference with 95% limits of agreement between weighed food records and image-based dietary assessment methods in assessing energy and macronutrients intake on weekdays compared with weekend days in free-living adults in Glasgow, UK, between January and August 2016

Method/nutrient	Weekdays (n = 80)		Weekend days (n = 80)		*P* value^a^
	Mean difference	Limits of agreement	Mean difference	Limits of agreement	
**FP**^b^					
Energy, kcal	–179	–962.4, 604.4	–235.2	–1,308.1, 837.7	0.438
Carbohydrates, g	–3.6	–298.9, 291.6	–20.5	–140.6, 99.5	0.348
Protein, g	–2.9	–45.4, 39.6	–11.0	–78.2, 56.3	0.077
Fat, g	–9.5	–56.3, 37.3	–12.5	–81.2, 56.3	0.497
**VR**^c^					
Energy, kcal	–56.5	–769.1, 656.2	–95.2	–1,092.0, 901.6	0.562
Carbohydrates, g	–6.6	–103.7, 90.4	–12.4	–122.4, 97.7	0.425
Protein, g	–0.9	–35.6, 33.8	–3.8	–61.4, 53.7	0.441
Fat, g	–1.6	–38.3, 35.1	–4.6	–62.6, 53.5	0.442

a *P* values were calculated by paired *t* test.

b FP = food photography.

c VR = video recording.

### Agreement Between WFR and VR in Estimating Energy Intake with Exclusion of Days with Missing Food Items

In 15 of the 160 (9.4%) VR days, some food items had not been recorded compared with the WFR diaries. When those days were excluded, estimated energy intake by VR was no longer statistically different from the WFR and the energy intake was underestimated by a mean error of –44 kcal (mean difference: *P* = 0.238; 95% limits of agreement –681.5 and 592.2 kcal) or a percentage estimation error of –2.9% (95% limits of agreement –42.5% and 36.6%) (Figure 3). The ability of VR to rank participants’ nutrient intake improved when analysis was repeated after exclusion of VR days with missing data (Table 2).

### Inter-Class Agreement

Table 5 displays the inter-class agreement within tertiles for energy and macronutrient intake using κ analysis. Weighted κ values of energy, carbohydrate, and protein intake showed moderate inter-class agreement for both methods. This was the case for estimates of fat intake for the FP method, whereas VR presented substantial agreement against WFR. The percentage of exact interclass agreement (percent of VR and FP assessments grouped in the same tertile) between the IBDA and WFR ranged from 61% to 69% with FP (energy: 69%, carbohydrates: 66%, protein: 65%, and fat: 61%) and from 67% to 76% with VR (energy: 70%, carbohydrates: 67%, protein: 71%, and fat 76%).

**Table 5:** Inter-class agreement by tertiles between weighed food records and image-based dietary assessment methods in assessing energy and macronutrients intake using weighted κ analyses in 80 free-living adults in Glasgow, UK, between January and August 2016

Nutrients	FP^a^∗∗∗	VR^b^∗∗∗
Energy, kcal	0.53	0.54
Carbohydrates, g	0.49	0.51
Protein, g	0.47	0.56
Fat, g	0.41	0.64

a FP = food photography.

b VR = video recording.

∗∗∗ *P* < 0.001.

### Methods Feasibly Evaluation

Participants' methods feasibility evaluation is presented in Table 6. IBDA methods were less time-consuming (*P* = 0.018), less boring (*P* = 0.015), more enjoyable (*P* < 0.001), and more practical (*P* = 0.002) than WFR (Table 6). No associations between participants’ demographics, BMI, or their profession and their feedback on method feasibility were found. Thirty-seven participants (46%) found that using FP had the same difficulty as using VR, whereas 42 (53%) reported that VR was more difficult. Fifty-six participants (70%) reported that the quantity of food they ate was the same as usual and most of them (75%) reported that their usual eating pattern was not changed during the recording period. Fifteen participants (19%) indicated that they did not record some food items they ate, mainly due to forgetting to record them (10%).

**Table 6:** Comparison of feasibility and practicality between weighed food records and image-based dietary assessment methods using Likert scales^a^ in 80 free-living adults in Glasgow, UK, between January and August 2016

Evaluation question	WFR^b^	IBDA^c^	*P* value^d^
	←*median (IQR*^e^*)*→		
**The method was**			
Unsociable	4.2 (1.5-5.9)	4.2 (1.2-6.5)	0.825
Difficult	2.6 (0.8-5.1)	2.1 (0.6-4.2)	0.098
Time consuming	5.3 (2.8-6.8)	4.6 (2.1-5.8)	0.018
Boring	4.6 (2.3-6.0)	3.8 (1.8-5.0)	0.015
Enjoyable	4.4 (2.3-5.6)	5.1 (4.1-6.5)	<0.001
Practical	4.4 (2.3-5.9)	5.5 (3.8-7.1)	0.002

a 0 (not at all) to 10 (very much).

b WFR = weighed food record.

c IBDA = image-based dietary assessment (including food photography and video recording).

d *P* values were calculated by paired *t* test.

e IQR = interquartile range (quartile 1 to 3).

Participants' quotes were grouped under similar themes. The major comments made by the participants were pertinent to the difficulty and the burden of weighing food items in WFR compared with the IBDA methods. Eleven participants (14%) reported that they enjoyed using the IBDA methods. However, 4 participants (5%) reported that using a camera was not that practical and they noted that using their mobile phone camera would be much easier to carry around. Three participants (3%) reported that they were embarrassed to weigh their meals and to record their voice in a video when other people or friends were present at mealtimes.

## Discussion

Accurate but also precise and practical methods of dietary intake assessment are required in public health nutrition surveys and by health care professionals to perform dietary assessment on individuals in clinical settings. The present study compared the performance of IBDA against the reference method of WFR. IBDA showed strong correlations in energy intake ranking, moderate inter-class agreement compared with the WFR, and strong inter-rater and intra-rater reliability. Collectively, these results suggest that IBDA and particularly the VR method might be suitable to use to rank individuals’ energy and nutrient intakes and might be used instead of WFR, when group estimates are required, such as in nutritional surveys. However, the use of IBDA for assessment of individuals’ intake is not recommended.

The average estimation bias of IBDA methods varied among the nutrients assessed and for the FP method exceeded 10% for energy intake. The VR method performed significantly better than the FP method in all nutrient and energy assessments, particularly after days with missing food items were omitted. However, for both methods and all nutrients assessed limits of agreement were wide. This suggests that their precision or error margin in assessment per individual may be considerable for some participants. Martin and colleagues,^45^ also reported a relatively small mean underestimation of –6.6% using FP compared with using WFR in estimating energy intake of free-living adults. Therefore, IBDA does not appear suitable to estimate nutrient intake in individuals, although any such judgment needs to be made in the context of the performance of alternative dietary assessment methods. With this in mind, the estimation bias reported by the use of IBDA in this study was comparable or lower than that of other mainstream methods of dietary assessment, including food frequency questionnaires, estimated weight food records and 24-hour dietary recalls.^46^, ^47^, ^48^ An advantage of IBDA compared with some of the conventional dietary assessment methods is that estimation of portion size is performed by a trained assessor. This can minimize portion-size estimation error, and over- or underestimation of intake compared with dietary assessment methods for which self-reporting and estimation of portion size are required by the participants.^49^^,^^50^ It is also usual practice in dietary assessment to record intake both during the week and on weekend days to account for estimation bias due to variation in eating habits or dining out on the weekend compared with weekdays, when meal patterns are more structured.^30^ However, no significant difference in estimation error was reported between recording dietary intake using the FR or the VR on weekday compared with weekend days, which is another advantage of these methods.

Among the IBDA methods tested in the current study, VR performed better than the FP method and particularly in the subset analysis when days with missing food items were excluded. Verbal information provided in the recorded videos on type of food consumed, meal recipes, cooking method, and food items difficult to recognize from photos alone (eg, spreads on bread) might explain the better performance of VR compared with the FP method. Similarly, in VR method, incomplete recording of food items consumed may have produced invalid comparisons with the WFR. In support of this, when videos containing missing food items were removed from comparative analysis, the mean estimation bias of VR was no longer statistically significant, the limits of agreement became narrower, and the overall performance of the method was much improved. Considering these findings and the additional burden introduced to the participants by obtaining food photographs and completing WFR at the same time, the authors of the current study recommend VR as the most accurate but also practical method to use.

Use of the IBDA methods were rated as more practical and enjoyable for participants, than use of WFR. Lassen and colleagues^51^ also reported that using a camera for estimating food intake was highly acceptable among adults participants. In the present study, some participants reported difficulty and inconvenience of food VR in public areas and this burden may have been further aggravated by applying all IBDA methods and WFR simultaneously.

The current study is not without its limitations. The recruited participants were mainly young adults and the results of this study may not apply to the performance of the IBDA methods in other age groups. Compared with older adults, young adults are probably much more comfortable using cameras and other digital technologies.^52^^,^^53^ Moreover, most of the participants in this study were students and were well educated, which is not representative of the general population in the United Kingdom. One would argue that because the participants were asked to record their food intake by applying the 3 methods simultaneously, this might result in transmitting the experience of recording from one method to another. However, the participants who captured the pictures and recorded the videos were not involved in any food intake recall or food portion size estimation, with all the collected data independently analyzed by a researcher. Applying all 3 methods simultaneously may have placed significant burden on participants compared with applying only 1 method a time; particularly when they were out of their homes. This might justify why some food items were not recorded with the IBDA methods, as well as the fact that 39% of the participants underreported their intake. As this study was comparing methods rather than assessing habitual intake, it was felt that the number of days recorded was not an important factor. In the current study, diet recording was applied during a short period and no fiduciary markers were used when capturing pictures and videos, as this would have placed extra burden on participants and might have reduced their compliance even further. To overcome this limitation, participants were trained to hold the camera at a certain distance from their meals. Whether estimation bias will increase or decrease during a longer period of diet recording needs to be explored as well. Another limitation is that a validated questionnaire was not available to use for the assessment of the feasibility and practicality of the IBDA methods. Moreover, evaluation of IBDA feasibility and practicality was not assessed separately for FP and VR, except for 1 question on method difficulty. In future research, such aspects should be explored separately for each of the IBDA methods and within the context of the characteristics of the population. The time taken to analyze the food images and videos captured was not formally recorded. However, the study researchers experienced considerable variation, depending on the meals consumed, including the number of individual food items in a meal. For certain items, such as packaged food and ready-to-eat meals, the type and portion size took a shorter time to be evaluated compared with other more complex food items, such as homemade recipes. Food consumed in public can differ from that consumed at home, which can potentially lead to bias and different performance of the IBDA when dining at home compared with when dining in public areas.

The main strength of the present study is that it evaluated participants' eating in free-living conditions and in various places instead of evaluating specific food items in a certain environment. Therefore, a large variety of foods and beverages were recorded and analyzed, which makes the results of this study more representative of the variable eating habits in a multicultural community, like in the United Kingdom. The advantage of using traditional digital camera over smartphone cameras is their better image resolution. In addition, by providing a digital camera, this ensured all photographs and videos were taken using the same device and any influence of the use of different cameras might have had on portion estimation was minimized.

## Conclusions

In young educated adults, IBDA methods may be useful tools for ranking dietary intake among populations and estimating their group intake, in particular VR, where the underestimation bias was <5% for energy and all macronutrients and was even lower when only complete data sets were used. However, the estimation error can vary considerably at assessment per individual and among the various nutrients. In addition, IBDA might remove the burden associated with estimation and recording of food portion sizes by the participants. Additional studies are required to test IBDA in different age groups, in clinical settings, during longer recording periods, and compared with other alternative methods of dietary assessment.

## Figures and Tables

**Figure 1:** Food photography and video recording instructions provided to participants to demonstrate how to record meals over 2 days using the camera provided in Glasgow, UK, between January and August 2016.

**PLEASE READ THROUGH THESE PAGES BEFORE STARTING FOOD INTAKE RECORDING** **Please record a video and take photographs of all food and drinks consumed at home and outside the home, for example, work, college, or restaurants, over 2 days (1 week day and 1 weekend day). It is very important that you do not change what you normally eat and drink just because you are recording your intake. Please keep to your usual food habits. Using the digital camera provided by the researcher, record your food before and after (leftovers) eating by photographing the same meals (single images and video) with a camera angle of 45 degrees and at a 1-arm distance from the object. Please add additional information when you record the video by describing the meal or the food with as much detail as possible. Don't include yourself in the recorded images and video.** **What to do while capturing a photograph?** Each time you eat use the camera provided to take a photograph of your served meals and then after eating take another photograph of any leftovers. Please take as many photographs as you wish at each time. If you are eating a prepacked meal please take pictures of the: a) brand, b) portion size, and c) food label. **What to do while recording the video?** Each time you eat please use the equipment provided to video your food. While recording, please describe the food you eat in as much detail as possible. Be as specific as you can. First, report the place and time of the meals (eg, Zizzi Italian restaurant in Glasgow at 7:30 pm). Then, describe the size of the plate and serving (eg, a medium serving of pasta with 3 tablespoons of Bolognese sauce on an average size shallow plate). Last, mention the cooking methods (fried, grilled, baked, etc) and any additions (fats, sugar/sweeteners, sauces, pepper, etc). **1. Homemade dishes** If you have eaten any homemade dishes, for example, chicken casserole, please name and record the name of the recipe, ingredients with amounts (including water or other fluids) for the whole recipe, the number of people the recipe serves, and the cooking method. Record in the video how much of the whole recipe you have eaten in the same way as for the other meals. **2. Take-away and eating out** If you have eaten take-away or dishes not prepared at home such as at a restaurant or a friend’s house, please record as much detail about the ingredients as you can, for example, vegetable curry containing chickpeas, aubergine, onion, and tomato. **3. Brand name** Please say the brand name (if known). Most packed foods will list a brand name, for example, Bird's Eye, Hovis, or supermarket’s own brands. **4. Labels/Wrappers** Labels are an important source of information for us. It helps us a great deal if you take a picture of the labels from foods, particularly of lesser-known food brands and also the labels of any supplements you take. **5. Portion sizes** For foods, quantity can be described in the video using: a. Use household measures, for example 1 teaspoon (tsp) of sugar, 2 thick slices of bread, ½ cup of gravy. b. Food labels, for example, 4-oz steak, 420-g tin of baked beans, 125-g pot of yogurt c. Number of items, for example, 4 fish fingers, 2 chicken nuggets, 1 Rich Tea biscuit d. The size of glass, cup, etc (eg, large glass) or the volume (eg, 300 mL). e. Volumes from labels (eg, 330 mL can of fizzy drink). **The researcher will show you examples of pictures and video footage that have been taken by different people. The examples will show you how we would like you to record your meals by the use of the camera provided.** **It only takes a few minutes for each eating occasion!**
